# Psychiatric mental health nurse practitioner student knowledge and perceptions of pharmacogenetic testing

**DOI:** 10.3389/fgene.2023.1281075

**Published:** 2023-10-13

**Authors:** Corrina M. Kaltenrieder, Michelle Marie White, Dennis J. Cheek

**Affiliations:** Harris College of Nursing and Health Sciences, Texas Christian University, Fort Worth, TX, United States

**Keywords:** pharmacogenetic testing, mental health, medication response, provider knowledge, graduate nursing

## Abstract

Psychotropic medications are typically prescribed in a trial-and-error fashion, and some providers are beginning to utilize pharmacogenetic testing (PGx) as a supplemental prescribing tool in treatment decision making. PGx testing shows potential in enhancing provider insights into personalized prescribing for patients by examining genetic information related to drug metabolism. Literature points to providers’ lack of knowledge in PGx interpretation as a main barrier, including psychiatric mental health nurse practitioners (PMHNPs). The aim of this study was to measure a difference, if any, in the knowledge and perceptions of PGx after implementation using a pre-post design. This study implemented an educational intervention on graduate nursing students (*n* = 15). Data were collected by using a pre- and post-interventional questionnaire. Results demonstrated a significant difference in findings related to students’ knowledge (*p* < 0.001), students’ skills related to pharmacogenetics, (*p* < 0.001), as well as students’ perceived ability to implement pharmacogenetics into their practice, (*p* = 0.028). The authors propose that the knowledge gained from the study demonstrates the importance of introducing PGx education into the PMHNP curricula and to prepare future PMHNPs to confidently utilize PGx in their clinical practice.

## Introduction

Nurse practitioners are assuming a greater majority of primary care for patients, which is changing the landscape of healthcare in the United States. The statistics demonstrate this, with an anticipation in growth of approximately 6.8% of new nurse practitioners in the United States by 2030, compared to only 1.1% of new physicians and 4.3% of new physician assistants (Auerbach et al., 2020). Moreover, it is anticipated that there will be a lack of specialist providers soon. Therefore, specialty care nurse practitioners, such as those who practice psychiatry, will play a bigger role in patient care and treatment planning (Coombs, 2015).

Providers in psychiatry treat a vast array of illnesses using psychotropics. Unfortunately, the efficacy of psychotropic medications is highly variable, and in the realm of psychiatry it is expected that treatment failure and unwanted side effects are inherent to pharmacotherapy (Riggs, 2020). Diagnoses such as Major Depressive Disorder (MDD) and Generalized Anxiety Disorder (GAD) are particularly difficult to treat, and usually require at least one drug trial before achieving a desirable response (Parish et al., 2023). Clinical experience and use of heuristics often aid the provider in deciding which medication to use for a given mental illness. However, burgeoning technology, such as pharmacogenetics testing (PGx), may be helpful for the provider for illnesses that may be treatment-resistant or in cases where the stakes are decidedly high, such as in those patients who are acutely suicidal and where time is of the most essence. As noted by Howes et al. (2022), up to 30% of patients suffering from treatment-resistant depression may attempt suicide in their lifetime.

As occurs with new technology, there has been some reluctance and lag in getting genetic testing into routine psychiatric clinical practice. In general, mental healthcare providers may be hesitant to rely on genetics for decision-making while others may be unaware of its utility. Without concrete data, it is unknown exactly why reluctance might exist. What can be surmised from the current literature is that the lack of standardization and provider knowledge of how to use PGx are the key issues (Thompson and Brooks, 2011).

In specific relation to nurse practitioners who specialize in mental health and psychiatry, the knowledge of PGx or the ability to utilize it in clinical practice is difficult to surmise given how young PGx is. What is known is that the call for nursing leadership in advancing genetic technology in the healthcare setting has been outlined in various publications (Prows and Saldaña, 2009). Importantly, those who work in advanced practice nursing must focus on showing the efficacy of testing in practice and explore how to facilitate policies and procedures related to implementing PGx in clinical practice to aid in the widespread adoption of PGx. More than a decade ago, the American Nurses Association published *Essentials of Genetic and Genomic Nursing, Competencies, Curricula Guidelines, and Outcome Indicators* (Consensus Panel, 2008), which recommended that nurses of all educational levels need to maintain pace with science as it evolves. Yet, research indicates that only a small percentage of nurses attend genetic/genomic continuing education, and some educational curricula lack this content (Thompson and Brooks, 2011). The literature demonstrates that targeted PGx educational sessions and how to interpret and utilize PGx guidelines in practice can improve genetics knowledge and bolster interest (St-Martin et al., 2017).

Provider knowledge of PGx has been noted as one potential barrier to its implementation in practice (Thompson and Brooks, 2011). For the current study, providers focus on advanced practice registered nurses. To date, there is a repository of clinical practice guidelines that is available for the application of PGx information; an example includes the Clinical Pharmacogenetics Implementation Consortium Guidelines (2021). Yet, while this information has been readily available for some time, the use of PGx in clinical nursing remains lacking (Burke et al., 2016).

PGx is a prescribing tool that can assist in personalizing care and decrease chances of unwanted side-effects. PGx guidelines include procedure recommendations, patient education, access to the most up-to-date evidence, and emerging science on genetic testing (Hicks et al., 2015). Exploration of future providers’ perceptions of PGx utilization in treatment planning and relevance to evidence-based practice lends insight into the feasibility of incorporating PGx into PMHNP curriculum.

The tailoring of treatments to an individual’s characteristics, needs, and preference is defined as precision medicine (Redekop and Mladsi, 2013). Pharmacogenetics is a form of precision medicine and explores the role of gene-drug interactions and inter-individual responses to a drug (Cheek et al., 2015). Differences in drug metabolism may manifest in different therapeutic effects, including untoward effects (Halter, 2017). The information gained from pharmacogenetic testing may provide healthcare practitioners a framework for decision-making when planning medication management and provide essential knowledge on how patients respond to specific drugs based on their pharmacogenetic profile.

Meta-analyses showed improved symptom remission when PGx was used in combination with provider expertise (Brown et al., 2021). Furthermore, the science of personalized prescribing is supported by evidence regarding the influence of several genetic variants on the pharmacokinetic actions of drugs. As it relates to psychotropic medications, many of these drugs are metabolized by the enzyme substrates CYP2D6 and CYP2C19 (Ivanov et al., 2022). PGx testing and patient genotypes demonstrated from testing may infer whether patients are intermediate, normal, or ultrarapid metabolizers (Hicks et al., 2015). With over 100 variants and subvariants of *CYP2D6*, personalized dosing and prescribing may be a difficult task without genetic testing (Gaedigk et al., 2018). Campos et al. (2022) discovered that differential metabolism of Selective Serotonin Reuptake Inhibitors (SSRIs) related to *CYP2C19* variants had a significant impact on drug tolerance and side effects (*p* = 0.002). In that study, intermediate or poor metabolizers of sertraline reported more side effects and intolerability than their rapid metabolizer counter parts (Campos et al., 2022).

In the current study, the authors aimed to assess if psychiatric mental health nurse practitioner (PMHNP) students’ knowledge of PGx was enhanced by the introduction of educational information and a guideline of PGx testing for serotonin reuptake inhibitors assessed by pre- and post-implementation measures given before the guideline and then after. Information gathered from PMHNP students provides a shift toward the use of PGx testing in a mental healthcare setting, an ultimate long-term goal of this project. Additionally, the information gleaned from this study can be used as a springboard to determine how the addition of PGx-related information into nursing graduate curricula may help to prepare future practitioners for clinical practice.

## Methods and materials

The current study received expedited review from the Texas Christian University (TCU) Compliance and Institutional Review Board (IRB), IRB# 2022-227. Participants were de-identified through convenience sampling. The university in which the study occurred is a small, private school. Data was examined through a focus on pre- and post-education intervention for a comparison with the participant serving as their own control. The survey was intended to measure differences in knowledge and perceptions of PGx after education on PGx.

To assess stakeholders’ knowledge and perceptions of PGx at baseline, the pre-implementation survey was disseminated to students in the Doctor of Nursing-PMHNP program. These students were recruited during a mandatory on-campus intensive educational session, as part of their graduate degree program. The post-survey taken after the education session was identical to the pre-survey. Students were either in the second or third year of the DNP-PMHNP program. No identifying information was used and surveys were voluntary.

Survey questions were adapted from a previous study survey by Weng et al. (2013) (2013). Questions were aimed at measuring changes in participant’s awareness, beliefs, attitudes, knowledge, skills, implementation, and system utilization of PGx (see Appendix). Due to time constraints, a pilot study and formal validation process was not possible. However, apart from the *Systems* domain, all other domains, and the question format mirror that of the evidenced-based survey by Weng et al. (2013). The authors added the *Systems* domain as the participants are not licensed providers but are registered nurses who may have observed PGx utilized in a patient care setting. Due to the varied nature of students’ backgrounds, some students may have had prior exposure to PGx in practice than others. We attempted to quantify baseline knowledge in the survey prior to our designated intervention.

Following the pre-survey, education on PGx result interpretation and barriers to utilization were provided for the student participants. The educational intervention and content were developed with a couple of educational videos, a PowerPoint, as well as walking students through how a provider would use PGx results in practice. To emphasize the scientific underpinnings of PGx and explain how pharmacogenetics can impact drug metabolism, students were introduced to a PGx guideline, the *Clinical Pharmacogenetics Implementation Consortium* (*CPIC*) *Guideline for CYP2D6 and CYP2C19 Genotypes and Dosing of Selective Serotonin Reuptake Inhibitors* (Hicks et al., 2015). Upon completion of the education session, there was an opportunity for discussion regarding barriers to PGx including insurance coverage, costs, ordering provider requirements, and medical and billing coding. Once discussions were closed, a post-survey identical to the pre-survey was provided to reassess stakeholder’s knowledge and perceptions of PGx post intervention.

### Analysis

Pre-and post-surveys assessed changes in students’ knowledge of how PGx is utilized in prescribing and changes in their comfort with utilizing PGx in their future practice. Survey questions were measured on a Likert scale, with a score closer to 1 indicating “strongly agree” and a score closer to 5 indicating “strongly disagree.” Data was analyzed using dependent *t*-tests to assess differences from baseline to post-intervention. Evaluating any changes in pre- and post-surveys after PGx education was necessary to conclude whether providing evidenced-based tools and education on PGx influenced the DNP-PMHNP students’ knowledge and perceptions of PGx as a prescribing tool.

### Inclusion/exclusion criteria

Inclusion criteria required participants to be active students currently enrolled in the DNP-PMHNP program. For participation in the survey, there were no specific requirements such as years completed in the program. To apply for the DNP-PMHNP program at TCU, prospective students are required to be bachelor’s prepared experienced registered nurses (RNs). Exclusionary criteria are any person not enrolled in a DNP-PMHNP program.

## Results

**FIGURE 1:**
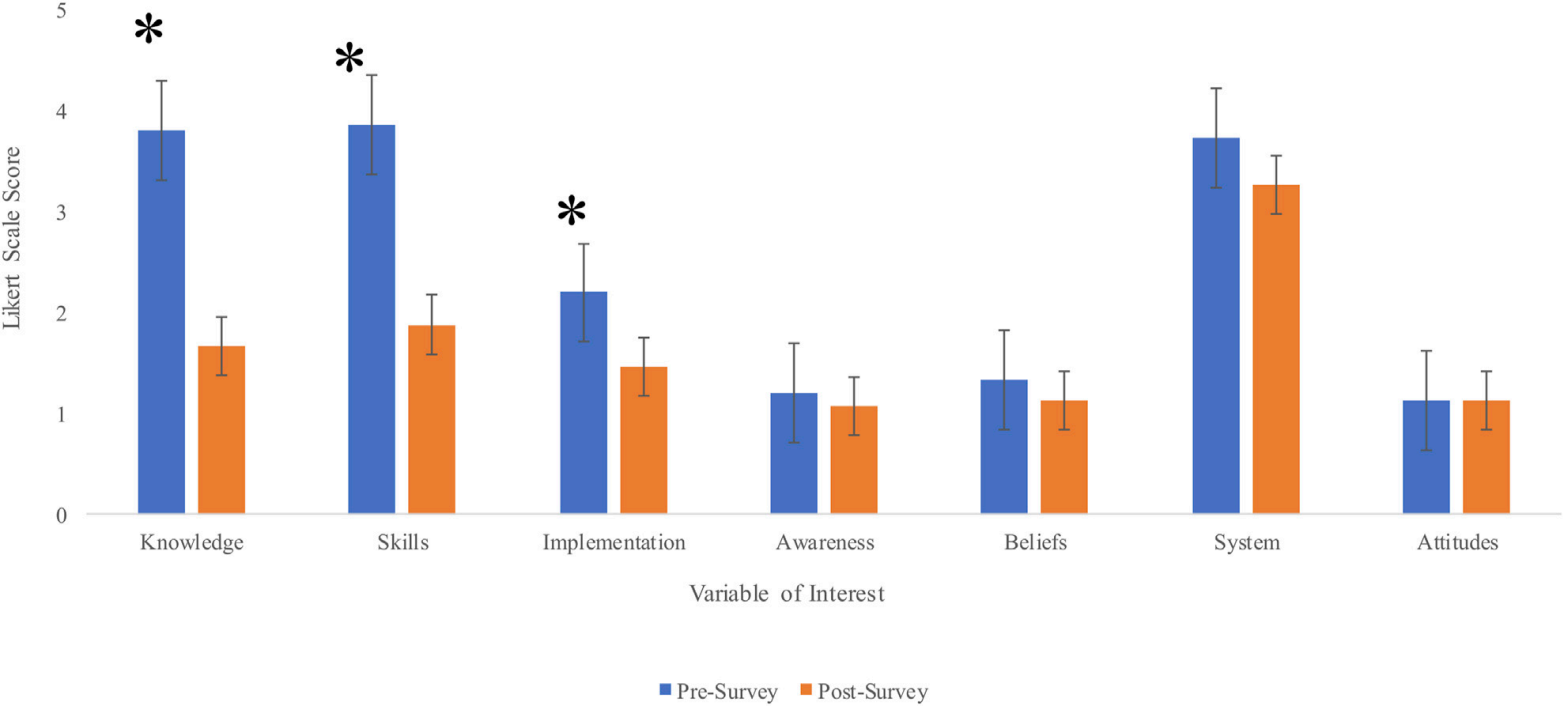
A comparison of pre- and post-survey results for the studied components. Note: Asterisks indicate significant differences in data from pre-to post-survey.

Results of the study were analyzed using Windows IBM SPSS version 27.0. There were 15 students in attendance, and 15 pre-survey and 15 post-surveys were completed. As shown in Figure 1, results demonstrated significant differences from pre-to post-testing in relation to students’ knowledge of PGx, *t*(15) = 6.959, *p* < .001, students’ skills related to PGx, *t*(15) = 6.959, *p* < .001, as well as students’ perceived ability to implement PGx into their practice, *t*(15) = 6.959, *p* = 0.028 (See Figure 1). Significant differences were not discovered in students’ awareness, *t*(15) = 1.468, *p* = *ns*, beliefs, *t*(15) = 1.871, *p* = *ns*, nor systems which is one’s experience observing PGx in the clinical environment, *t*(15) = 1.240, *p* = *ns*. Interestingly, attitudes towards promotion of PGx showed no significant differences, *t*(15) = 1.1333, *p = ns*.

## Discussion

The results of the current study demonstrate the importance of dissemination of new knowledge and maintaining a sharp understanding of the state of the art, specifically in the setting of healthcare. Interestingly, between pre- and post-implementation of PGx education, students’ perception of their knowledge, skills, and ability to implement PGx evidence into practice significantly changed. While the field of PGx research can be opaque to new learners, education can serve to break down barriers that future providers may feel in using PGx in their clinical judgement and routine.

Variables of interest that did not demonstrate change from pre- to post-implementation included awareness, beliefs, and systems-related conceptualization of PGx. This may be because some students have had exposure to PGx concepts in their academic careers or have observed providers use PGx in the clinical environment. Additionally, it is not doubtful that belief systems related to certain scientific techniques may be deeply ingrained and resistant to change. While this is speculative at best, it is impossible to state for certainty why belief systems may remain unchanged. The results from the current study add to the current literature that relates to nurses’ knowledge base of PGx, specifically, those studying psychiatry and mental health. Other studies have reported similar results in improvements in concepts such as attitudes and knowledge, although in different specialties such as oncology and public health (Dodson, 2018; Zureigat et al., 2022), and researchers continue to note the importance of further contributions to this area of PGx science and nurse knowledge (Laaksonen et al., 2022).

## Limitations

The limitations of the current study include that the participant sample size was small (*n* = 15) and taken at a small, private school. Also, as participants were de-identified, and therefore demographic information needed to better understand any sociocultural differences in relation to the data acquired was excluded. Also, for this study dependent *t*-tests were used and with an small sample size, consequential low power is possible. However, the results of the current study provide a platform that will hopefully enable a broader delving into this subject matter across other educational formats and media.

## Conclusion and future work

The current research lends credence to the implementation of PGx material into graduate nursing coursework. The results of the project provide a platform that will hopefully enable a broader delving into this subject matter across other educational formats and media. The culmination of additional research will hopefully bear fruit in demonstrating the utility of PGx as a new technology to aid in the treatment of patients suffering from mental illness. Future recommendations include assessing more novice nurses in the field of psychiatry to target those individuals who may have never been exposed to graduate-level information related to PGx testing. Furthermore, incorporating PGx knowledge and skills in the standards of graduate nurse curriculum will prepare advanced practice nurses to educate patients on PGx and utilize PGx confidently in their practice.

## Data Availability

The raw data supporting the conclusion of this article will be made available by the authors, without undue reservation.

## Appendix

**TABLE A1:** Pre and post survey.

	Strongly agree	Somewhat agree	Neither agree nor disagree	Somewhat disagree	Strongly disagree
Awareness	1	2	3	4	5
I have heard of pharmacogenetic testing or related terms, such as pharmacogenomics, metabolizer status (Phenotype), and alleles					
Beliefs	1	2	3	4	5
I believe that the use of the best available genetic tests can assist in improving the quality of patient care					
Attitudes	1	2	3	4	5
I support the promotion of the implementation of genetic tests to improve patient outcomes					
Knowledge	1	2	3	4	5
I have sufficient knowledge to implement or help implement genetic testing to guide care in my future role as a PMHNP.					
Skills	1	2	3	4	5
I possess sufficient skills to implement or help implement genetic testing to guide care in my future PMHNP role					
Implementation: I can access relevant literature and evidence-based guidelines to resolve clinical questions about genetic testing and apply the findings to clinical decision making after critical appraisal	1	2	3	4	5
System	1	2	3	4	5
Within my organization I have observed providers order genetic testing to guide care in their practice					
